# Usutu virus infections among blood donors, Austria, July and August 2017 – Raising awareness for diagnostic challenges

**DOI:** 10.2807/1560-7917.ES.2017.22.41.17-00644

**Published:** 2017-10-12

**Authors:** Tamás Bakonyi, Christof Jungbauer, Stephan W. Aberle, Jolanta Kolodziejek, Katharina Dimmel, Karin Stiasny, Franz Allerberger, Norbert Nowotny

**Affiliations:** 1 Viral Zoonoses, Emerging and Vector-Borne Infections Group, Institute of Virology, University of Veterinary Medicine Vienna, Vienna, Austria; 2 Department of Microbiology and Infectious Diseases, University of Veterinary Medicine, Budapest, Hungary; 3 These authors contributed equally to this article and share first authorship; 4 Austrian Red Cross, Blood Service for Vienna, Lower Austria and Burgenland, Vienna, Austria; 5 Center for Virology, Medical University of Vienna, Vienna, Austria; 6 Austrian Agency for Health and Food Safety (AGES), Vienna, Austria; 7 Department of Basic Medical Sciences, College of Medicine, Mohammed Bin Rashid University of Medicine and Health Sciences, Dubai, United Arab Emirates

**Keywords:** Usutu virus, West Nile virus, flavivirus, *Flaviviridae*, blood-borne infections, blood donor, diagnosis, Austria

## Abstract

Between July and August 2017, seven of 12,047 blood donations from eastern Austria, reacted positive to West Nile virus (WNV) in the cobas test (Roche). Follow-up investigations revealed Usutu virus (USUV) nucleic acid in six of these. Retrospective analyses of four blood donors diagnosed as WNV-infected in 2016 showed one USUV positive. Blood transfusion services and public health authorities in USUV-endemic areas should be aware of a possible increase of human USUV infections.

In 2014, the Austrian Red Cross, Blood Service for Vienna, Lower Austria and Burgenland, initiated regular screening of all blood donated between 1 June and 30 November each year for West Nile virus (WNV) by a nucleic acid test (NAT). Positive samples have been found each year since [1]. In 2017, several positive samples were identified, however, WNV could not be confirmed by other molecular tests; this prompted us to conduct more detailed molecular analyses of these samples.

## Processing of blood samples

Samples of all blood donations collected from 1 June onwards by the Blood Service for Vienna, Lower Austria and Burgenland of the Austrian Red Cross during the ongoing WNV transmission season 2017 were sent to the German Red Cross, Blood Service for Baden-Württemberg-Hessen in Frankfurt, Germany, where they were screened in minipools of 19 samples using the automated NAT test on the cobas 8800 system (cobas WNV assay; Roche, Rotkreuz, Switzerland). As of 11 October 2017, a total of seven of 12,047 blood donations collected between 24 July and 25 August 2017 reacted positive. Positive samples were further evaluated at the Austrian National Reference Laboratory for Arbovirus Infections, the Center for Virology of the Medical University of Vienna, as well as at the Institute of Virology, University of Veterinary Medicine Vienna. WNV NAT-positive blood donors were notified and relevant demographical and epidemiological information was obtained from them. WNV NAT-screening positive samples were retested by WNV- and Usutu virus (USUV)-specific RT- and RT-qPCR assays as well as by conventional RT-PCR tests amplifying a wide range of flaviviruses [2-4]. While one blood donation was confirmed as containing WNV nucleic acid, the six others were negative by WNV-specific RT-qPCR but positive by USUV-specific RT-qPCR. The threshold cycle (Ct) values were between 34.7 and 38.3. Also, conventional RT-PCRs resulted in amplification products of the expected sizes, which turned out to be USUV by sequence analyses. Retrospective analyses of four blood donors diagnosed as WNV-infected in 2016 among 70,864 tested over the whole testing period (1 June to 30 November) showed one USUV positive sample. Moreover, no USUV-nucleic acid positive blood donation was found among a total of 74,677 donations tested in 2015.

## Description of the Usutu virus-positive blood donors

The seven USUV-positive donors were between 20 and 60 years of age, and consisted of six men and one woman. They all originated from the Austrian federal states of Vienna, Lower Austria and Burgenland, respectively. Six of them could be interviewed, and only one reported a temporary stay abroad (in Sicily) one to two weeks before blood donation. None of the donors reported clinical symptoms, neither in the four weeks before nor at subsequent controls after blood donation. Also the WNV-confirmed donor in 2017 was asymptomatic and had acquired the infection in Lower Austria.

## Phylogenetic analysis of the Usutu virus sequences detected

**Figure 1:**
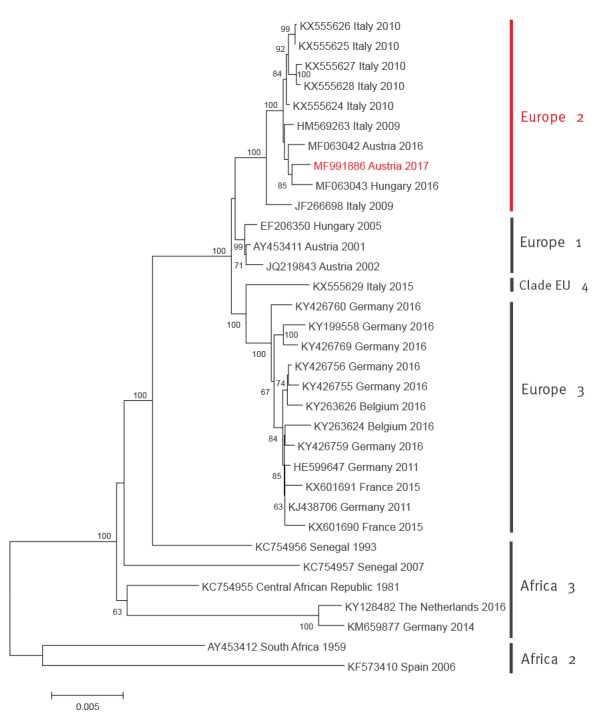
Phylogenetic analysis of selected complete coding Usutu virus sequences together with that recovered from a blood donor in Austria, 2017

**Figure 2:**
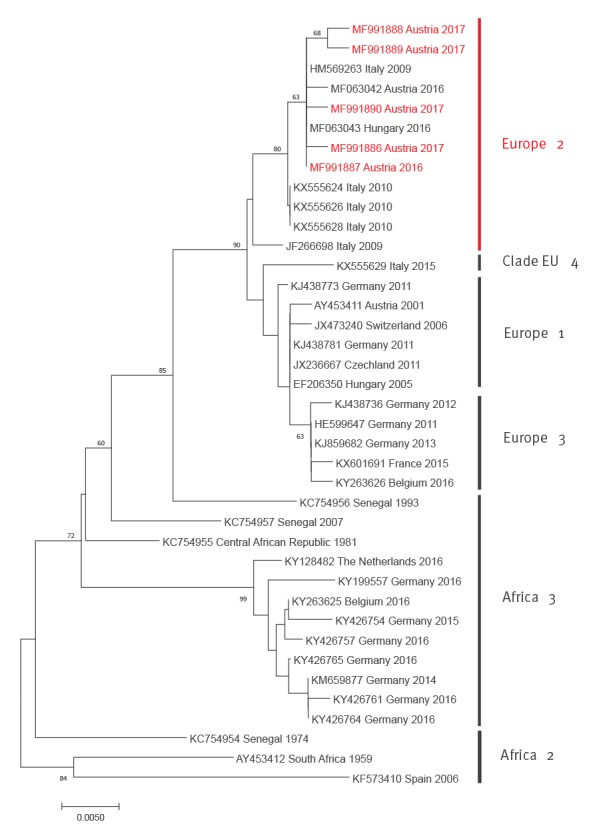
Phylogenetic analysis of selected partial NS5 protein coding and 3’UTR nt Usutu virus sequences together with those recovered from blood donors in Austria, 2016 and 2017

From the first NAT-positive blood sample in 2017 the complete coding sequence was established by primer-walking, Sanger sequencing of the amplification products and compiling the partial sequences to the complete coding sequence [5]. From the other 2017 NAT-positive samples and the single USUV-nucleic acid-positive blood donor specimen from 2016 partial sequences were generated within the NS5–3'UTR region and another region within the NS5 gene. Phylogenetic trees were established by the neighbour-joining method within the molecular evolutionary genetics analysis (MEGA)7 programme, for complete coding sequences as well as for partial NS5–3'UTR sequences as described previously [6]. In both phylogenetic analyses, based on complete coding and partial NS5–3'UTR sequences, all Austrian blood-donor-derived sequences cluster within the ‘Europe 2’ genetic lineage of USUV. They are closely related with each other and with Italian avian- and human-derived viruses from 2009 and 2010, including strain Bologna 2009 [7-9], and with recent (2016) bird-derived viruses from Austria and Hungary [6] (Figures 1 and 2).

Sequences are labelled by codes containing the GenBank accession number, country of origin, and year of sample collection. The sequence derived from the Austrian blood donor in 2017 described in this paper is highlighted in red font. Vertical bars on the right indicate USUV genetic lineages as suggested by Cadar et al. [18] and Calzolari et al. [26]. The phylogram was generated with neighbour-joining statistical method; bootstrap percentage values of 1,000 replicates ≥ 60% are displayed. The horizontal bar on the left represents the genetic distance. The highly divergent USUV strain KC754958 (Central African Republic 1969), which represents the ‘Africa 1’ genetic lineage, was omitted in order to increase the resolution of the phylogram.

The phylogenetic tree is based on partial NS5 protein coding and 3’UTR nt sequences (between nt positions 10,161 and 10,720 referring to the Usutu virus (USUV) genome sequence NC_006551). Sequences are labelled by codes containing the GenBank accession number, country of origin, and year of sample collection. The sequences from Austrian blood donors described in this paper are highlighted in red font. Vertical bars on the right indicate USUV genetic lineages as suggested by Cadar et al. [18] and Calzolari et al. [26]). The phylogram was generated with neighbour-joining statistical method; bootstrap percentage values of 1,000 replicates ≥ 60% are displayed. Horizontal bar on the left represents the genetic distance. The highly divergent USUV strain KC754958 (Central African Republic 1969), which represents the ‘Africa 1’ genetic lineage, was omitted in order to increase the resolution of the phylogram.

The sequences determined during this study were deposited in GenBank under accession numbers MF991886 (coding-complete sequence) and MF991887–90 (partial NS5–3'UTR sequences).

## Discussion

USUV, an African, mosquito-borne flavivirus [10], emerged in Vienna and its surroundings in 2001, where it caused considerable die-off of birds, mainly Eurasian blackbirds (*Turdus merula*) [2]. However, its emergence in Europe dates back to at least 1996, when an episode of wild bird mortality was observed in the Tuscany region of Italy [3]. USUV-associated bird mortality was subsequently found in several European countries [11-17]. In 2016, widespread USUV activity was reported in wild birds in Belgium, France, Germany and the Netherlands [18] as well as in Austria (in the same area as 2001−2006) and Hungary [6].

Only two USUV isolations from humans have been reported in Africa: one in 1981 from a patient with fever and rash in the Central African Republic, and the other in 2004 from a child with fever and jaundice in Burkina Faso [19].

In Austria, investigations in USUV-endemic areas in and south of Vienna during August and September 2003, when the virus was active in wild birds, revealed USUV-specific nucleic acid in the blood of a young man with rash, and USUV-neutralizing antibodies in 54 of 208 individuals tested [20].

In 2009 USUV nucleic acid was detected in two critically ill, immunocompromised persons in Italy: one patient with B-cell lymphoma who developed meningoencephalitis [21], and another patient who developed neurological disease after receiving an orthotopic liver transplant [7,8]. Meanwhile the complete coding sequence of the latter USUV strain (designated Bologna 2009) was determined and, in an attempt to identify genetic markers potentially associated with severe illness, compared with other USUVs [9].

In subsequent years, USUV-specific antibodies were detected in healthy blood donors as well as in patients with neurological symptoms in Italy, Germany and Croatia (summarised in [9] and [22]). Screening of 13,023 blood donations from the University Hospital Aachen, Germany, in 2016 – during the simultaneous widespread USUV epidemic in birds in western and central Europe [6,18] – revealed one flavivirus-RNA-positive sample with the cobas TaqScreen WNV test, which was identified as USUV [23]. In a recent study conducted between 2008 and 2011 in the Modena region of Italy, which involved 915 patients with or without neurological impairments, the percentage of USUV RNA- and antibody-positive samples was significantly higher than that of WNV, raising the issue on the potential of USUV to cause human neuroinvasive disease [22]. The deleterious effect of USUV on human neural cells was also shown experimentally [24]. 

In Austria, blood donors have to answer an extended medical questionnaire before donation. Several questions refer to visits to countries endemic for pathogens transmissible by substances of human origin including WNV. Travellers returning from WNV-endemic regions have either to be screened by WNV NAT or have to be deferred for 28 days after their return from an affected area.

The goal of this communication is to raise awareness of blood transfusion services and public health authorities in countries, which have reported USUV infections: (i) of an increase of USUV detections in blood donations in eastern Austria in 2017 compared with the two previous years, and (ii) of the possibility that USUV infections may be misdiagnosed as WNV, due to tests not distinguishing between these two viruses.

The latter is actually not a real problem because any flavivirus (including both WNV and USUV) infected blood donation must be discarded; nonetheless the diagnosis should be verified/falsified by virus-specific follow-up molecular tests to precisely identify the causative agent. Aetiological diagnosis might also reveal other flaviviruses such as Zika virus, dengue virus, etc. The minimum sensitivity of the Roche cobas WNV assay is approximately 13 copies/mL in a pooled sample, which corresponds to 250 copies of WNV RNA/mL for each individual blood donation (Health Canada standard). The virus loads in the USUV positive samples were low, therefore not all samples were uniformly positive in all conventional and RT-qPCRs applied, consequently only five sequences could be included in the NS5–3'UTR phylogenetic analysis (Figure 2). Despite the observed cross-reactivity, the Roche cobas WNV test is a sensitive and very valuable screening test for flavivirus nucleic acid in blood samples. Cross-reactivity was also found in another WNV screening test [8].

The phylogenetic analyses demonstrated a close genetic relationship of the Austrian blood donor-derived USUV sequences with the recent bird-derived Austrian and Hungarian sequences [6], suggesting that the same viruses are capable of infecting both animals and humans. The same is true for the German Aachen 2016 blood donor-derived sequence, which clusters together with bird-derived sequences from the same region in the ‘Europe 3’ cluster [23]. The Austrian coding-complete sequence derived from a healthy blood donor in 2017 is almost identical to the Bologna 2009 sequence isolated from a critically ill patient with neurological symptoms [7-9] – 10,769/10,792 nt identities and only two amino acid differences (Asp_770_Asn and Lys_2520_Arg). This might suggest that host factors such as age, comorbidities, and immunosuppression, and not necessarily genetic markers of the infecting virus, influence the clinical outcome of a human USUV infection. It is also worth mentioning that none of the Austrian USUV-positive blood donors showed any clinical symptoms.

Although USUV is a potential human pathogen, there are no specific regulations on screening blood donations for this virus. According to the general rules, only clinically healthy people are allowed to donate blood. However, as described above, the USUV-positive blood donors did not show any symptoms before and after donation. Although WNV and USUV endemic areas in Europe largely overlap [25], there are some countries (particularly Belgium, Germany, the Netherlands and Switzerland) where USUV is endemic, but autochthonous WNV infections have not been reported so far. Blood transfusions are frequently applied to immunosuppressed or critically ill patients so there is a potential risk for development of nosocomial USUV-associated neurological disease in such patients, in USUV endemic countries that do not screen for flaviviruses. Therefore we propose that extension of flavivirus screening of blood donations might be taken into consideration by USUV-endemic European countries.

In order to raise awareness and allow timely actions, we intentionally wrote this paper at this point in time when the WNV and USUV transmission seasons are still ongoing, which, depending on weather conditions, may last in central Europe until the end of October.
